# A plant 35S CaMV promoter induces long-term expression of luciferase in Atlantic salmon

**DOI:** 10.1038/srep25096

**Published:** 2016-04-26

**Authors:** Tore Seternes, Tom C. Tonheim, Anne I. Myhr, Roy A. Dalmo

**Affiliations:** 1 University of Tromsø, Faculty of Biosciences, Fisheries & Economics, Norwegian College of Fishery Science, 9037 Tromsø, Norway; 2 Genøk – Centre for Biosafety, The Science Park, Postbox 6418, 9294 Tromsø, Norway

## Abstract

The long-term persistence and activity of a naked plasmid DNA (pGL3-35S) containing a *luc* gene (reporter gene) controlled by a plant 35S CaMV promoter was studied in Atlantic salmon (*Salmo salar* L.) after injection. Atlantic salmon (mean weight 70 grams) were injected intramuscularly with 100 μg of plasmid DNA. Blood, different tissues and organs were sampled at different time points up to day 535 after injection. Southern blot analysis suggested the presence of extra-chromosomally open circular, linear and supercoiled topoforms of pGL3-35S at day 150 after injection. At day 536 open circular and supercoiled topoforms were detected. Luciferase activity was detected at the injection site up to 536 days post-injection of pGL3-35S, where it peaked at day 150 and decreased to approximately 17% of its maximum activity by day 536. Our study demonstrated that a plasmid containing the 35S promoter was able to induce expression of a reporter gene/protein in fish *in vivo* and that the plasmid DNA persisted for a prolonged time after intramuscular injection.

In most of the genetically modified (GM) plants commercialised or undergoing field trials, the 35S Cauliflower Mosaic Virus (CaMV) promoter is used to secure transgene expression. The 35S CaMV promoter is generally considered to be a strong constitutive promoter^1^, and it facilitates high level of RNA transcription in a wide variety of plants, including plants well outside the host range of the virus^2^. Previous studies have shown that the 35S CaMV promoter drives transgene expression in *Escherichia coli*^3^, yeast^4^,^5^,^6^ and mushroom^7^. Whether the 35S CaMV promoter confers activity with respect to gene expression in lower and higher vertebrates such as fish and mammals has been a matter of debate within the scientific community^8^. To date, results from transgene expression experiments using the 35S CaMV promoter have been reported for *in vitro* systems, such as human embryonic kidney cells^9^, Chinese hamster ovary cells^10^, human enterocyte-like cells^11^,^12^, *Xenopus* oocytes^13^ and several fish cell lines^14^. These latter reports conclude that the 35S CaMV promoter is active in several eukaryotic cell lines after transfection – achieved by use of transfection reagents. The 35S CaMV promoter has been shown to drive the expression of green fluorescent protein for only two weeks after being injected intramuscularly in rainbow trout^14^. However, it is not known whether a 35S CaMV promoter would remain active in fish in a long-term perspective. As such, the research hypothesis is that a naked plasmid DNA containing a *luc* gene (reporter gene) controlled by 35S CaMV promoter can express the transgenic luciferase more that for one year *in vivo* after being injected to Atlantic salmon.

## Material and Methods

Luciferase Assay System (Cat # E1501) and QuantiLum^®^ Recombinant luciferase (Cat # E1701) were obtained from Promega (Madison, WI, USA). Bio-Rad RC DC Protein Assay kit II (Cat # 500-0122) was purchased from Bio-Rad Laboratories (Hercules, CA, USA). All other chemicals, unless stated, were purchased from Sigma Chemical Co (St. Louis, MO, USA).

### Fish

Seventy non-vaccinated Atlantic salmon (*Salmo salar* L.), with a mean weight of 70 g, were obtained from the Tromsø Aquaculture station (University of Tromsø – The Arctic University of Norway and NOFIMA, Norway). The experimental studies were conducted in running natural fresh water at temperatures 0–12 °C (temperature of inlet fresh water), and injections were performed at 8 °C. The fish were adapted to the test conditions at least one week before use and fed a standard diet during the experimental period. The Norwegian Animal Research Authority approved all experimental protocols involving live fish to be in compliance with the Animal Welfare Act (https://www.regjeringen.no/en/dokumenter/animal-welfare-act/id571188/), the current approval was given the identifier ID3880. We confirm that all experiments were performed in accordance with relevant guidelines and regulations given by the Norwegian Animal Research Authority.

### Plasmid DNA

The commercial (promoterless) pGL3-Basic vector (Promega) was used to construct a pDNA with the firefly (*Photinus pyralis*) luciferase reporter gene downstream of the 35S CaMV promoter (558 bp); pGL3-35S (approx. 4.8 kbp), as described earlier^11^. GenØk (Centre for Biosafety (Tromsø, Norway) kindly provided this plasmid vector. The plasmid pLG3-Basic contained no other promoter or enhancer sequences (technical information provided by Promega), and did not contain any PvuII cleavage site. The pGL3-Control vector with the SV40 promoter upstream of the *luc* gene (pGL3-SV40) and the promoterless pGL3-Basic vector with the *luc* gene (approx. 4.8 kpb) were all purchased from Promega. All pDNAs were propagated by transformations of *Escherichia coli* DH5α grown in Luria Bertani medium with 100 μg ml^−1^ ampicillin and isolated using Qiagen Plasmid Mega Kit (Qiagen, GmbH, Hilden, Germany) according to the manufacturer’s recommendation.

### Plasmid DNA injection

One hundred micrograms of pDNA [pGL3-35S, pGL3-SV40 and pGL3-Basic (plasmid control)] corresponding to approximately 1.4 mg kg^−1^ body weight were injected intramuscularly in 100 μl 0.02 M phosphate buffered saline (PBS) (iso-osmotic, pH 7.75), with a 25-gauge needle. The intramuscular (i.m) injection was performed in the right epaxial muscle below the dorsal fin in a perpendicular manner. Thirty fish were i.m injected with 100 μl of PBS, and served as negative controls fish for each treatment group.

### Evaluation of 35S-promoter efficiency

Five fish from each treatment groups receiving pGL3-35S, pGL3-SV40, pGL3-Basic or PBS were killed at day 7, and liver, kidney, spleen, heart, gills, muscle, anterior and posterior intestine and tissue surrounding the injection site were harvested. Tissues were frozen directly in liquid nitrogen and stored at −86 °C for 1 day before performing luciferase activity assay – by following the protocol by the manufacturer. The luciferase was detected using a Luminoscan Ascent® micro plate illuminometer (Thermo Electron Oy, Vantaa, Finland). The relative light units (RLU) were normalized to the protein concentrations in the sample determined by the Bio-Rad RC DC protein assay (Bio-Rad Laboratories). The output results (chemiluminescence) were highly dependent on the colour intensity of the tissues. To avoid false positives and negatives, standard curves were made on each tissue where a known amount of recombinant luciferase (QuantiLum^®^ Recombinant luciferase; Promega) was added to different dilution of tissue homogenates.

### Time-course expression of luciferase in muscle tissue

Five fish from each of the treatment groups receiving pGL3-35S or PBS were killed 7, 30, 70, 150 and 536 days after i.m injection. Tissue surrounding the injection site was harvested, frozen directly in liquid nitrogen and stored at −86 °C for 1 day before performing the luciferase activity assay.

### Quantification of luciferase activity in tissues of Atlantic salmon

Tissues were prepared according to Promega’s “tissue homogenate” protocol^15^, and the luciferase activity assay has been described elsewhere^16^. In brief, pooled samples were pre-processed for luciferase activities, the relative emitting light (chemiluminescence) units (RLU) were normalized to the protein concentrations using measurements from Bio-Rad RC DC assay (Bio-Rad Lab.). To avoid false positives and negatives, standard curves were made on different tissues where a known amount of recombinant luciferase (QuantiLum®Recombinant luciferase; Promega) was added to different dilutions of tissue homogenates.

### Persistence and conformational analysis of re-isolated pGL3-35S

Five fish from each of the treatment receiving pGL3-35S were killed 7, 30, 70, 150 and 536 days after i.m injection (same fish as described in the paragraph “*Time-course expression of luciferase in muscle tissue*”). At day 7, 30, 70, 150 and 536 the liver, kidney, spleen, heart, gills, muscle, anterior and posterior intestine and tissue surrounding the injection site were harvested for Southern blot analysis. Samples from PBS injected fish (N = 3) served as controls. Total DNA was extracted from tissue samples using the E.Z.N.A.®Tissue DNA Kit II (Omega Bio-Tek, Doraville, GA, USA) according to the manufacturer’s recommendation. Ten micrograms of total DNA from the injection site of pGL3-35S and PBS injected fish were digested with *Pst* I (Promega) or *Pvu* II (Promega) and run on a 0.7% agarose gel, blotted and visualised by enzyme-linked chemiluminescence using a Digoxigenin (DIG)-labelled PCR probe. The DIG labelled PCR probe was constructed using the PCR DIG Probe Synthesis Kit from Roche (Roche Diagnostics, Mannheim, Germany), forward primer 5′-CAAATCATTCCGGATACTGCG-3′and reverse primer 5′-CCCGGTTTATCATCCCCCT-3′, amplifying a 397 bp region of the *luc* gene. Primers were designed using the Primer Express software (version 2.0; Applied Biosystems) and synthesised by Operon Biotechnologies Inc. (Eurofins Genomics) (Huntsville, AL, USA).

### Statistics

The experiments were carried out once, the number of replicate fish of each experimental treatment groups at each sampling time point were five (N = 5). The Statistical analysis was performed using two-tailed, paired Student’s T-test.

## Results

### Quantification of luciferase activity in Atlantic salmon tissues

**Figure 1:**
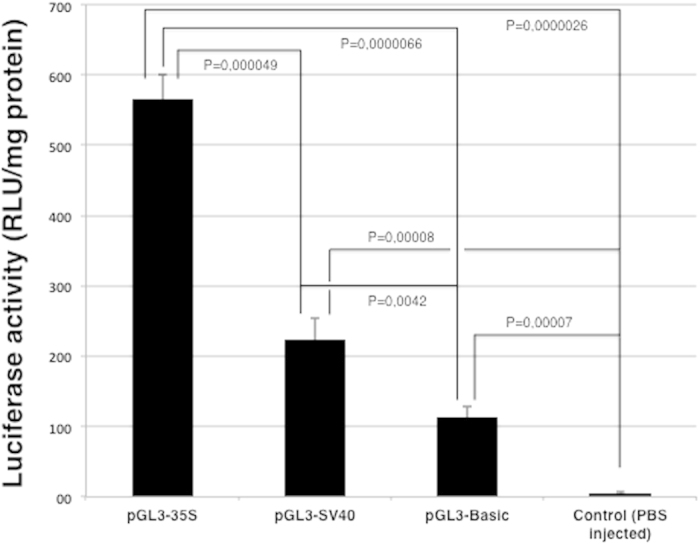
Luminescence quantification of luciferase activity in Atlantic salmon muscle tissue 7 days after intramuscular injection of pDNA (pGL3-35S, pGL3-SV40, pGL3-Basic (promoterless)) or PBS (control). Each bar represents the mean luciferase activity of five fish per treatment group, and error bars represent S.D. The measured luciferase activity was normalised for total protein content in muscle. P-values are inserted.

To enable a quantitative assessment of the efficacy of the 35S CaMV promoter, a luciferase reporter gene with the 35S CaMV promoter upstream was used (pGL3-35S). As a positive control pDNA containing the SV40 promoter (pGL3-SV40) was included in the experiments, whereas the promoter-less pDNA (pGL3-Basic) and samples from fish injected with PBS served as negative controls. In this first experiment, the fish were killed at day 7, since previous studies have shown a high degree of reporter gene expression at this time-point using several different other promoters in different animal models^15^,^16^,^17^,^18^. Luciferase activity in samples obtained from the site of injection was confirmed in all groups of fish that received pGL3-35S and pGL3-SV40 (Fig. 1). No luciferase activity was found in samples from the other tissues and organs sampled or in the control fish injected with PBS.

The background luciferase activity in muscle controls (injected with PBS) was 5.1 ± 1.4 RLU mg^−1^ protein (±S.D). The luciferase activities from muscle samples obtained from fish injected wit pGL3-Basic, pGL3-SV40 and pGL3-35S were approximately 22, 43 and 110 times higher compared to the average level detected in fish injected with the PBS, respectively. The expression of luciferase in control muscle tissue (PBS injected fish) was statistically significantly lower than for pGL3-Basic (p = 0,00007), pGL3-SV40 (p = 0,00008) and pGL3-35S (p = 0,0000026). The difference in expression of luciferase between muscle tissues obtained from fish injected with pGL3-SV40 (p = 0,0042) and pGL3-35S (p = 0,0000066) were statistically significant higher than for pGL3-Basic (Fig. 1 and Supplementary data).

### Longevity of luciferase expression

**Figure 2:**
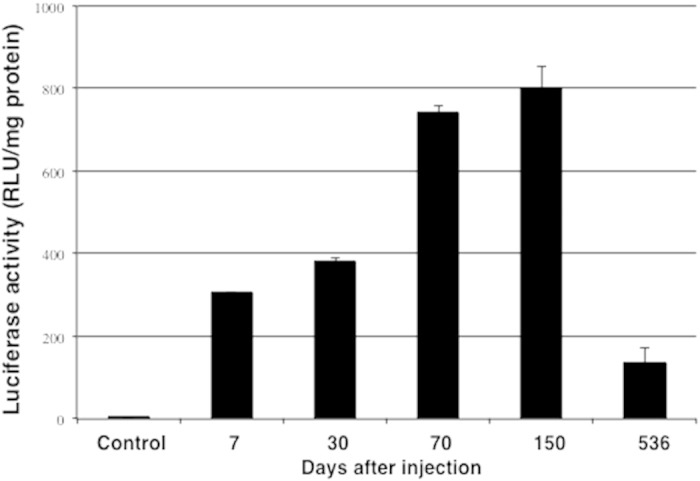
Luminescence quantification of luciferase activity in Atlantic salmon muscle tissue 7, 30, 70, 150 and 536 days after intramuscular injection of pGL3-35S. PBS injected fish served as control. Each bar represents the mean luciferase activity of five fish and T-bars represent the S.D. The measured luciferase activity was normalised for total protein (mg).

Luciferase activity was not detected anywhere else than in muscle tissue (injection site) at the first sampling time-point at day 7 post-injection. Consequently, examination for long-term luciferase expression was subsequently performed on the muscle tissues only. Luciferase activity was detected at the injection site up to 536 days post-injection of pGL3-35S (Fig. 2), where it peaked at day 150 and decreased to approximately 17% of its maximum activity by day 536 (Fig. 2).

### Conformation of re-isolated pGL3-35S

**Figure 3:**
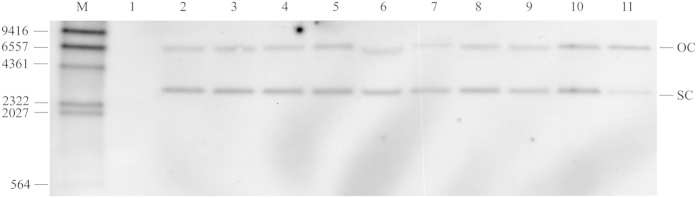
Southern blot showed extra-chromosomally open circular and supercoiled topoforms of pGL3-35S in different tissues on day 7 days in the muscle of Atlantic salmon. M: DIG labelled DNA Molecular Weight Marker II (Roche), OC: open relaxed topoform, SC: supercoiled topoform, 1: kidney control DNA, 2: liver, 3: kidney, 4: spleen, 5: heart, 6: gill, 7: muscle, 8: anterior intestine, 9: posterior intestine, 10: injection site, 11: 100 pg of pGL3-35S. DNA from samples of five fish from each treatment groups were mixed to yield samples for the Southern blot experiment.

**Figure 4:**
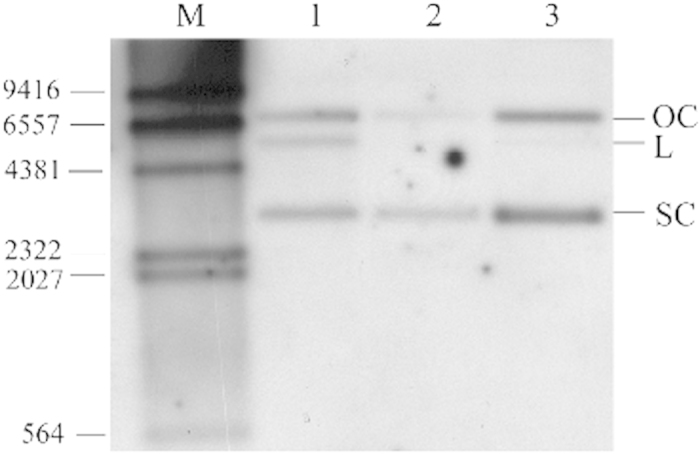
Southern blot analysis on DNA from the injection site after intramuscular injection of pGL3-35S in Atlantic salmon. Samples shown were obtained 150 and 536 days after injection. M: DIG-labelled DNA Molecular Weight Marker II (Roche), OC: open circular topoform, L: linear topoform, SC: supercoiled topoform, 1: day 150, 2: day 536, 3: 100 pg of pGL3-35S. DNA from samples of five fish from each treatment groups were mixed to yield samples for the Southern blot experiment.

To determine the conformation of the resident pGL3-35S at day 7, total DNA from all samples were digested with the endonuclease *Pvu* II. Subsequent Southern blot analysis suggested the presence of two extra-chromosomally located different topoforms of pGL3-35S in the liver, kidney, spleen, heart, gills, muscle, anterior- and posterior intestine and at the site of injection 7 days post-injection (Fig. 3). These topoforms were presumably open circular (OC) (approx. 6,5 kbp) and supercoiled form (SC) (approx. 3 kpb), respectively. No pGL3-35S was detected in control kidney sample from fish injected with PBS (lane 1). The presence of pGL3-35S in the injection site at 14, 70, 150 and 536 days post-injection was also determined by Southern blot analysis. Linear pGL3-35S was present in the muscle tissue at the injection site 14, 70, 150 and 536 days after injection –evaluated by Southern blot of samples priory digested with *Pst* I (data not shown). Furthermore, total DNA collected at day 150 and 536 from the site of injection was digested with the restriction enzyme *Pvu* II. Southern blot analysis suggested extra-chromosomally open circular, linear and supercoiled topoforms of pGL3-35S at day 150 (Fig. 4; lane 1). A sample retrieved from the stock plasmid preparation (pGL3-35S) contained mostly open circular and supercoiled topoforms, and minor amount of linearized topoform (Fig. 4; lane 3). However, only the open circular and supercoiled topoforms were detected at day 536 (Fig. 4; lane 2). Samples obtained from control fish contained no pDNA.

## Discussion

The measured enzymatic activity of plasmid-encoded luciferase at the injection site seven days after injection (cf. Fig. 1) indicated that the pGL3-35S plasmid containing the CaMV promoter indeed induced expression *in vivo,* as the SV40 promoter (pGL3-SV40) did. This is the first evidence that a plasmid containing the 35S CaMV promoter is able to give expression of a reporter gene/protein in a vertebrate species *in vivo*.

We were then interested to study the long-term expression of luciferase (enzyme) at the injection site using this construct containing the 35S CaMV promoter. Interestingly, expression of luciferase activity was detected in the muscle tissue at the injection site even 536 days after injection. This is comparable with previous findings where luciferase under the control of a CMV promoter also promoted expression of luciferase for 536 days^19^ and also comparable to observation made in the glass catfish^20^.

We cannot offer any explanation why the promoter- and enhancer-less pGL3 Basic induced expression of luciferase at the injection site 7 days after plasmid injection. However, the product fact sheet provided by the manufacturer (Promega) (https://no.promega.com/~/media/files/resources/protocols/technical%20manuals/0/pgl3%20luciferase%20reporter%20vectors%20protocol.pdf) states that PGL3 Basic may induce certain level of luciferase protein expression albeit this plasmid vector do not contain any promotor or enhancer elements. We have also found similar result, in a control experiment, where the pGL3-Basic was able to induce a certain luciferase activity; 0.7% relative to R70pRomiLuc containing a CMV promoter, after being transfected into NIH3T3 cells *in vitro*. This support the finding that pGL3-Basic may induce luciferase expression at the injection site – albeit at a low level.

Previous to this study we, however, found that the measured luciferase (by luminometer) activity was highly dependent on the inherent tissue colour. As a consequence, we extrapolated the luciferase activity from a standard curve made on control tissue homogenates with added recombinant luciferase. The quenching by tissue colours was higher in blood-rich tissues and tissues with strong colour, while it was close to zero in muscle tissue that did not contain much colour. This extrapolation would add a small uncertainty to the data set, but the interpretation of the results is not changed.

The next research questions were whether this expression of luciferase activity was related to presence of intact plasmid DNA at the injection site. Seven days after injection, both open circular and supercoiled plasmid DNA (pGL3-35S) were found in all tissues and organs analysed. The stock solution of pGL3-35S also contained both supercoiled and open circular conformations (result not shown). The tissue processing and DNA isolation steps prior to conformational analysis have in previous study not been found to cause any strand breaks in the DNA^21^. In the current study, a tiny portion of pDNA may be resident/hidden at the injection site, being responsible for the long-term expression of the transgene luciferase – probably inside muscle cells though no proofs exist for this in fish or in mammalian species.

Analysis of samples obtained at time-points up to 535 days post injection also showed presence of open circular and supercoiled topoforms (+presumably linearized ones), but only at the injection site. Tonheim *et al*.^19^,^21^ also reported the presence of similar topoforms in different tissues and organs together with the injection site after CMV-containing plasmid DNA was injected in A. salmon. Although plasmid DNA was present in many different tissues at day 7, no luciferase activity was detected except at the injection site. These results are supported by the findings shown by Tonheim *et al*.^16^,^21^.This finding is in contrast with previous study that described luciferase expression, under CMV, and activity in distally localised tissues after i.m. injection been shown for rainbow trout where plasmid DNA encoding luciferase under CMV promoter control was injected^22^.

When pDNA (DNA vaccine) is injected into muscle, several events are initiated: 1) Uptake of pDNA by cells at the administration site, 2) pDNA remains extracellularly in the administration site, 3) Degradation of pDNA by endonucleases at the administration site, and 4) distribution of pDNA by blood, cells and lymph to various tissues^23^.

This study has confirmed for the first time in any vertebrate that a S35 CaMV promoter is able to drive expression of a transgene, and that the duration of transgene production is at least 1.5 years *in vivo.*

## Additional Information

**How to cite this article**: Seternes, T. *et al*. A plant 35S CaMV promoter induces long-term expression of luciferase in Atlantic salmon. *Sci. Rep.* **6**, 25096; doi: 10.1038/srep25096 (2016).

## Supplementary Material

Supplementary Information
